# Estimating Age-Specific Immunity and Force of Infection of Varicella Zoster Virus in Norway Using Mixture Models

**DOI:** 10.1371/journal.pone.0163636

**Published:** 2016-09-30

**Authors:** Emanuele Del Fava, Grazina Rimseliene, Elmira Flem, Birgitte Freiesleben de Blasio, Gianpaolo Scalia Tomba, Piero Manfredi

**Affiliations:** 1 Carlo F. Dondena Centre for Research on Social Dynamics and Public Policy, Bocconi University, Milan, Italy; 2 Department of Vaccines, Norwegian Institute of Public Health, Oslo, Norway; 3 Oslo Centre for Biostatistics and Epidemiology, University of Oslo, Oslo, Norway; 4 Department of Infectious Disease Epidemiology, Norwegian Institute of Public Health, Oslo, Norway; 5 Department of Mathematics, University of Rome Tor Vergata, Rome, Italy; 6 Department of Economics and Management, University of Pisa, Pisa, Italy; Hokkaido University Graduate School of Medicine, JAPAN

## Abstract

This study applies mixture modelling to examine age-specific immunity to varicella zoster virus (VZV) infection in Norway based on the first large-scale serological study in the general population. We estimated the seropositive proportions at different ages and calculated the underlying force of infection by using a sample of 2103 residual sera obtained from patients seeking primary and hospital care. A rapid increase in the VZV-associated immunity is observed in the first years of life with 63% of children being immune by age 5. The increase in the immunity levels slows down thereafter, with a large proportion of adults still susceptible by age 20 (around 14.5%), thus at risk of serious sequelae of varicella infection. The corresponding force of infection peaks during the preschool period, subsequently declines to a minimum between ages 10 and 20 years, and afterwards moderately increases to reach a plateau lasting throughout the childbearing period. In comparison with the traditional cut-off approach, mixture modelling used the whole data without producing any inconclusive cases, led to an unbiased classification of individuals between susceptible and immune, and provided a smoother immune profile by age. These findings represent an important step towards any decision about the introduction of varicella vaccination in Norway, as they are a primary input for mathematical transmission models aimed at evaluating potential vaccination scenarios.

## Introduction

Varicella zoster virus (VZV) is a DNA virus belonging to family *Herpesviridae*, which is transmitted by airborne droplets and direct contact [1]. VZV is responsible for two clinical manifestations, namely varicella (chickenpox) and herpes zoster (shingles) (HZ). Varicella is a highly transmissible infection occurring early in childhood, with 90% of European children becoming immune by age 12 in the absence of vaccination [2]. Varicella is usually benign with complications occurring in 2%-6% of cases [3]. After recovery, the virus remains latent in the sensory ganglia and can reactivate at later ages causing HZ, a skin disease yielding serious morbidity [4]. Although HZ immunology and pathogenesis are still poorly understood, there is agreement that reactivation occurs when cell-mediated immunity (CMI) declines [5]. The latter is based on the work of Hope-Simpson [6], who also hypothesized that CMI might be boosted by re-exposure to VZV, referred to as the “exogenous boosting” hypothesis (EBH). Some evidence supporting this hypothesis has gradually accumulated [7–9], albeit the importance of natural boosting in development of HZ remains not well understood [10].

Safe and effective varicella vaccines have been available since 1970s [11], and are recommended by WHO [12]. Currently, due to the unclear impact of varicella vaccination on the epidemiology of HZ, only a few European countries have introduced varicella vaccination into their national programmes [13]. Indeed, all published mathematical models of VZV transmission predicted an increase in the incidence of HZ over several decades following mass varicella vaccination [8,14–17], due to the decreasing incidence of natural boosting. However, empirical evidence on this phenomenon in countries currently using universal varicella vaccination is still controversial [18–20]. Thus, countries like Norway that are not vaccinating against varicella should first carefully assess their local VZV epidemiology as a necessary step towards a thorough evaluation of possible varicella and HZ vaccination scenarios, before making any decision about immunisation programmes.

Traditionally, serological studies use antibody levels to define proportions of susceptible and immune individuals by applying a set of fixed cut-offs. Individuals with antibody levels higher than the upper cut-off are classified as seropositive, those with antibody levels below the lower cut-off are classified as seronegative, while antibody levels in between the two cut-offs are considered as inconclusive. For serological purposes, these individuals are usually retested or discarded from the analysis. For its diagnostic nature, the use of cut-offs results in a low test sensitivity, i.e., the ability of detecting true positive cases, thereby making this approach less appropriate for epidemiological studies, but also more prone to misclassification errors and to biased seroprevalence estimates [21].

Conversely, mixture modelling has been used as a more accurate approach to optimally categorise between susceptible and immune individuals [21–25], because it fully uses serological data and provides the following important by-products: i) the reclassification of the individual antibody levels between susceptible and immune, including those considered as inconclusive by the cut-off approach; ii) the age-specific proportions seropositive in the sample on the basis of the mixture reclassification [22,23]; iii) the unbiased estimates of the population age-specific seroprevalence and force of infection (FOI), with related precision, assuming either parametric or non-parametric models for these two functions [24,25].

To this end, for the first time, we applied the mixture modelling approach to VZV antibody levels data from Norway, where VZV seroprevalence was recently examined by the traditional cut-off approach [26]. Hence, the purpose of this work was to obtain unbiased estimates of pre-vaccination VZV seroprevalence and associated FOI based on the unbiased categorisation of individuals into susceptible and immune driven by mixture modelling. These estimates provided insights into the natural history of VZV infection in Norway, laying the ground for the future development of dynamic transmission models of varicella and HZ and for the assessment of the potential impact of different immunisation strategies against the two infections.

## Materials and Methods

### Samples and laboratory methods

VZV seroprevalence data were collected in a cross-sectional study using anonymised residual sera from patients of all ages (age range: 0–92 years) seeking both primary and hospital care in Norway [26]. For each patient, the following information was available: sex, year of birth and sample collection date (these two pieces of information were used to calculate the patient’s age), county of residence, and laboratory name. A total of 2103 residual serum samples (males: 52%) were collected from all Norwegian counties in 2006, 2007, 2008, 2011 and 2014 (samples collected during the 2009–2010 influenza pandemic were excluded). Allocation of samples to age strata was designed to allow comparison with other European studies [2]. Roughly 100 samples were randomly selected for each of the following age groups: 1-year bands between 0 and 9 years, 5-years bands between 10 and 49 years, 10-years bands between 50 and 69 years, and 100 samples for the age group 70 years and higher. All serological tests for VZV-specific IgG were performed using a commercial ELISA assay according to the manufacturer’s guidelines [26]. This assay allows results to be expressed using a set of two fixed cut-off points. In particular, samples with a corrected optical density (OD) >0.2 at 450nm were classified as seropositive, samples with OD <0.1 were classified as seronegative, and samples with OD between 0.1 and 0.2 were classified as inconclusive. Inconclusive samples were not re-tested. The Regional Committee for Medical and Health Research Ethics (Oslo, Norway) approved the study, as well as an exemption from patient’s consent to use residual sera. The anonymised samples were obtained from the biobank at the Norwegian Institute of Public Health following the internal procedures and the approval of the same Regional Committee. Further details and results based on the traditional cut-off approach are reported in [26].

### Mixture modelling

We consider the population to be at demographic and epidemiological equilibrium. For infections conferring lifetime immunity such as varicella, the age-specific seroprevalence *π*(*a*) represents the expected proportion of immune individuals at exact age *a* in a given population, and it is the complement of the susceptible proportion *x*(*a*), i.e., *π*(*a*) = 1−*x*(*a*). The latter satisfies the equation *x*′(*a*) = −*λ*(*a*)*x*(*a*), where the relative speed of decline *λ*(*a*) is the age-specific force of infection (FOI), i.e., the per-capita rate at which a susceptible individual aged *a* acquires varicella infection. We estimate the functions *π*(*a*) and *λ*(*a*) directly from the individual antibody levels data using finite mixture models [27].

We assume that the serological sample is drawn from a population consisting of only two subpopulations, the susceptible one and the immune one. This is because no universal varicella vaccination programme is in place in Norway; thereby, data do not present a high heterogeneity in the antibody levels of immune individuals, which is typically common for post-vaccination serology [28,29]. The resulting density function *g* for the antibody level distribution, among individuals aged *a*, can be represented as a mixture distribution of two unknown densities of the form $g\left(Y_{i}\left(a\right)\right)=\sum_{k=1}^{2}\pi_{k}\left(a\right)f\left(Y_{i}|\xi_{k}\right)$, where the antibody level of individual *i*, Y_i_, is measured by a logarithmic transformation of the individual OD values, corrected in order to deal with negative values, i.e., Y_i_ = log_10_(OD_i_+1), *a* denotes chronological age, *f*(*Y*_*i*_|*ξ*_*k*_), *k* = 1, 2, are the mixture components, *π*_*k*_(*a*) are the age-dependent mixture weights of the two components, and, finally, *ξ*_*k*_, *k* = 1, 2, are the vectors of parameters to estimate for each of the components [27]. In particular, the age-dependent weight of the immune component, *π*_2_(*a*), represents the population seroprevalence [30].

Given the current lack of knowledge in the data generating process underlying the antibody level data, different density functions can be taken to model the data. An obvious choice is the normal distribution, which is a reasonable assumption mostly for the susceptible component [31]. However, it has been reported that, for some infections, the immune component might be characterised by skewness (with a long right tail), thus other distributions that allow for it ought to be considered [22,23]. A viable alternative is then the skew normal distribution, which generalises the normal distribution by allowing for skewness through a specific parameter [32,33]. In case this parameter took value zero, the skew normal distribution would then reduce to the normal distribution. Hence, since data inspection reveals a substantial degree of positive skewness in the immune component, we opted for the skew normal distribution. The mixture model has therefore the following formula: $g\left(Y_{i}\right)=\sum_{k=1}^{2}\pi_{k}\left(a\right)SN\left(Y_{i}|\mu_{k},\sigma_{k}^{2},\alpha_{k}\right)$, where *μ*_*k*_, $\sigma_{k}^{2}$, *a*_*k*_, *k* = 1, 2 denote the mean, the variance, and the skewness parameters of the two mixture components, respectively. A positive (negative) value of *α* implies a right- (left-) skewed distribution. The parameters *μ*_*k*_, $\sigma_{k}^{2}$, *a*_*k*_, *k* = 1, 2 are assumed to be age-independent, which means that the antibody level distribution per component is assumed to be the same for all the groups, making the model highly parsimonious.

In our analysis, we stratify the sample by one-year age groups up to age 60, while samples from people 60+ years are merged into one group. In this way, we assess changes in seroprevalence in more detail and reduce the risk of merging groups with possibly different serological patterns. Moreover, we exclude from the analysis all the individuals aged less than one year because of the presence of maternal antibodies up to 6 months of age [34] and the absence of information on age in months in this group [26].

Model estimation and inference are carried out by using Bayesian Markov Chain Monte Carlo (MCMC) methods. More specifically, we combine a prior distribution for the unknown parameters with the data likelihood, as it occurs within the Bayesian setting, and then we compute the posterior distribution of the unknown parameters using MCMC methods through Gibbs sampling [35].

Further details on the estimation of the mixture parameters are reported in the S1 Text.

#### Classifying individual antibody levels as susceptible or immune

Each observation is assigned either to the immune or the susceptible component with probability equal to the seroprevalence and its complement, respectively. For this purpose, we introduce a latent age-dependent classification variable, *Z*_*i*_(*a*), which represents the unknown infection status of individual with age *a* [35] and has the following Bernoulli distribution:

$$Z_{i}\left(a\right)=\{\begin{array}{ccc} 1 & \text{with probability }\pi \left(a\right) & \text{seropositive},\\ 0 & \text{with probability }1-\pi \left(a\right) & \text{seronegative}, \end{array}$$

where *π*(*a*) is the population seroprevalence [30]. This means that the seroprevalence function governs the assignment of individual antibody levels to the components. The binary classification variable *Z*_*i*_(*a*) mimics the binary variable of the observed serological status, as determined by the cut-off approach. However, as it is not given with the data, but rather estimated through the analysis, it enters the model as a latent variable.

#### Computing the proportions seropositive by age

Based on the estimates of the classification variable, ${\bar{Z}}_{i}\left(a\right)$, we calculate the proportions seropositive by age in the sample as the number of individuals assigned to the immune component $\left({\bar{Z}}_{i}\left(a\right)=1\right)$ in each age group divided by the total number of individuals in the same age group [29]. We note that the proportions seropositive, unlike the seroprevalence, are not subject to any constraint, as they reflect a specific realisation of the event of being seropositive to VZV in the sample.

#### Estimating population seroprevalence and FOI by age

We assume the age-specific seroprevalence to follow a nonparametric model, under the constraint of being a monotonically non-decreasing function of age, which guarantees in turn that the FOI is a non-negative function of age [25].

For its estimation under the Bayesian framework, we choose a beta prior distribution for each age group *j*, namely *π*_*j*_~*Beta*(*α*_*j*_, *β*_*j*_), under the monotonically non-decreasing constraint *π*_*j*−1_ ≤ *π*_*j*_ ≤ *π*_*j*+1_, which is then combined with the data to provide the posterior distribution of the age-specific seroprevalence [36].

Finally, the FOI *λ*_*j*_ in each age group is estimated using the formula $\;\lambda_{j}=\pi_{j}^{\prime }/(1-\pi_{j})≅\left[\left(\pi_{j+1}-\pi_{j-1}\right)/2\right]/\left(1-\pi_{j}\right)$.

## Results

**Fig 1:**
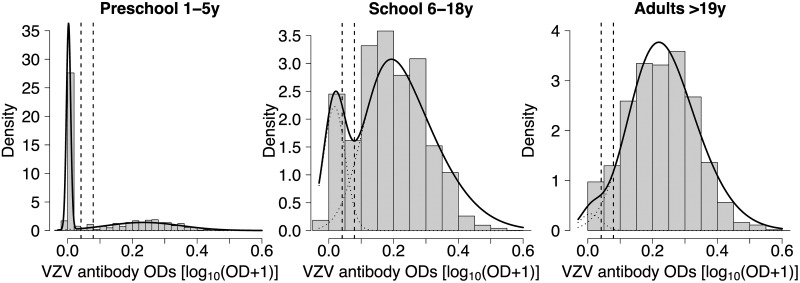
Distribution of antibodies to VZV by age in Norway. Histogram of the log-transformed antibody optical densities (log_10_(OD+1)) against VZV by broad age group (preschool: 1–5 years; school: 6–18 years; adults: 19 years and more), with over imposed the corresponding density function estimated by the mixture model, for each component (dotted line), and for all the data (solid line). The vertical dashed lines represent the manufacturer’s fixed cut-offs and the data included between the two lines are usually classified as inconclusive under the conventional cut-off approach.

We assessed whether the mixture model provided a good fit to the data by comparing the histograms of the antibody levels by broad age groups (preschool: 1–5 years, school: 6–18 years, adults: 19 years and more) with the corresponding distributions predicted by the mixture model (Fig 1). Based on visual inspection, the fit appears very satisfactory for both the immune and the susceptible components. Close correspondence between the mixture distribution and the histogram of data is important because it implies a precise allocation of individuals to the serological status groups, and hence an accurate estimate of seroprevalence. The good fit is encouraging, considering that the adopted model is quite parsimonious, as it postulates the same distribution of antibodies in all age groups. Antibody levels in the susceptible subpopulation are always concentrated around zero, while the antibodies in the immune subpopulation are characterised by a high degree of heterogeneity, likely due to variability in time elapsed since infection, given that subsequent exposures do not seem to affect humoral antibody levels [9]. The estimated value of the skewness parameter for the immune component is positive and equal to 2.19 (95% CI: 1.67, 2.38), while for the susceptible component is not significantly different from 0 (95% CI: -0.18, 0.20). These values are confirmed by the data (Fig 1), which show a little degree of positive skewness in the immune component. Finally, if we look at the way cases that were classified as inconclusive by the cut-off approach (encompassed by the two vertical dashed lines in Fig 1) are reclassified under the mixture modelling approach, we notice that, for all age groups except school-age children, most of the inconclusive cases are usually reclassified as seropositive cases. This shows how the mixture model approach generally yields a larger test sensitivity compared to the fixed cut-off approach.

**Fig 2:**
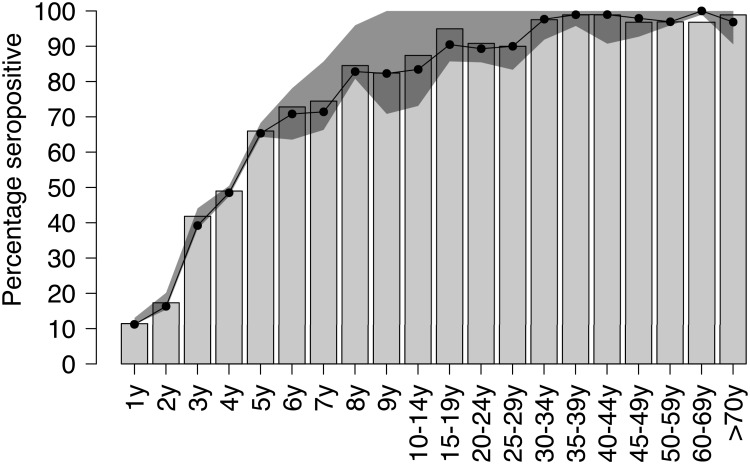
Proportions VZV-positive individuals in Norway. Age-specific proportions seropositive determined using the fixed cut-off (grey bars) and the mixture model (black dots). For the latter ones, we also reported the 95% pointwise credible intervals (grey area).

The proportions seropositive estimated in the initial analysis under the conventional cut-off approach [26] are compared to those estimated with the mixture model (Fig 2). We note that the proportions obtained from the mixture model tend to be equal than those obtained from the cut-off in the younger age groups, mostly at 4 and 5 years of age, where varicella circulation is more rapid; afterwards, among school age children, the mixture model suggests a smoother, but generally lower, immune profile compared to the cut-off-based estimates. The lower immunity profile among school age children found by the mixture model with respect to the cut-off method (Fig 2) may be explained with the reclassification of inconclusive cases within this age group into seronegative cases (Fig 1). Finally, the proportions seropositive under the two approaches overlap for ages between 25 and 60 years of age, as among the elderly, older than 60 years, we can see again some differences between the two approaches.

**Fig 3:**
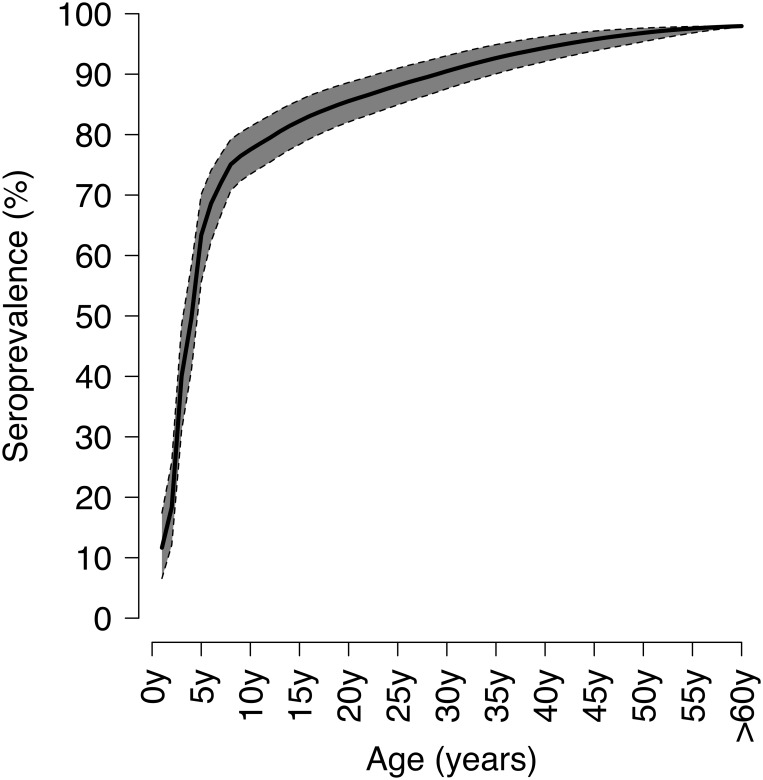
Seroprevalence of VZV in Norway. Posterior mean of the age-specific VZV seroprevalence in Norway (with 95% CI), determined using the mixture model.

**Fig 4:**
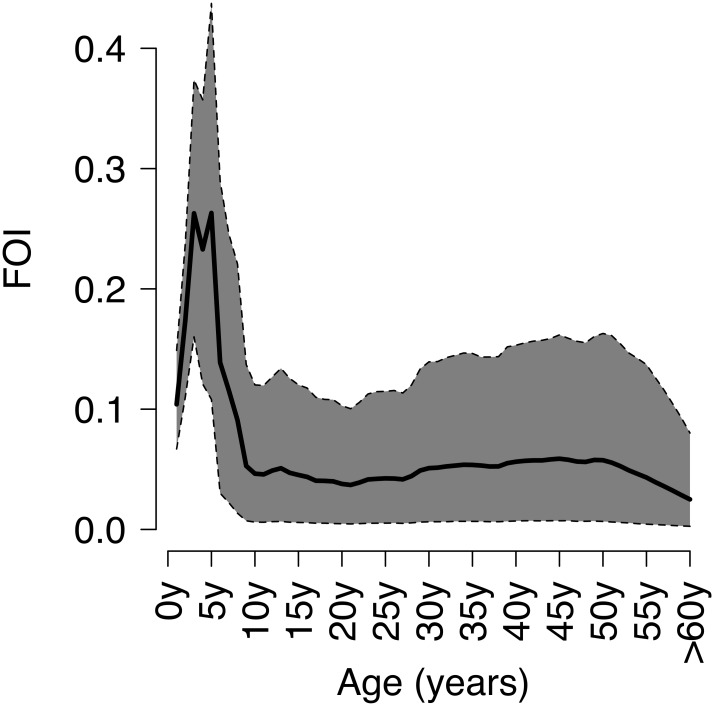
FOI of VZV in Norway. Posterior mean of the age-specific VZV FOI in Norway (with 95% CI), determined using the mixture model.

The age-specific seroprevalence estimated by the mixture model (Fig 3) reveals a smooth profile, increasing initially very rapidly (up to 63% at age 5 and 75% at age 8), as reflected by the high levels of the FOI (Fig 4). After 8 years of age, the seroprevalence profile continues to increase, but at a much smaller, almost constant, rate, so that quite substantial proportions of individuals are still susceptible during the childbearing period, with 85.5% of individuals being immune at 20 years and just about 90.5% at 30 years of age. In particular, 45.8% of these susceptible adults in their childbearing period are women, who might thus be at risk of contracting varicella during pregnancy. Eventually, we find that 1.6% of individuals aged 60 years or more are still susceptible to VZV (Fig 3). After the aforementioned peak in the preschool period (between 3 and 5 years of age), the corresponding FOI (Fig 4) shows a rapid decline to a minimum, followed by a moderate, but prolonged, increase to a plateau lasting for the entire childbearing period (ages 20–45 years), before sharply declining thereafter. S1 Table reports the estimates of the age-specific FOI with the respective 95% CIs.

## Discussion

In this paper we applied mixture modelling to data on antibody levels collected in the first large-scale serological survey of VZV in Norway to obtain unbiased estimates of pre-vaccination VZV seroprevalence and FOI [25]. The good fit of the mixture model to the antibody levels data suggests that the method is successful in assigning individuals to their correct serological status group and therefore in correctly estimating the seroprevalence. Due to a reclassification of all cases, including those excluded for being classified as inconclusive by the cut-off approach, we obtained a generally smoother set of estimates of the immune proportions at each age compared to the traditional cut-off approach. The derived best estimate of the seroprevalence profile showed a rapid initial increase in VZV seropositivity, with 63% of individuals being already immune by 5 years of age. This growth however slows down already during the first years of primary school, and further decelerates after 8 years of age, showing from that age and onwards a rather small, almost constant, increasing rate. During the childbearing period, a substantial proportion of individuals is still susceptible to varicella: in particular, the predicted seroprevalence is about 85% at 20 years of age and 90% at 30 years of age. In comparison with other countries [2,37–39], our results indicate that the acquisition of immunity against varicella in Norway in the first years of life (prior to the beginning of the primary school) happens more rapidly than in Southern Europe, where generally a slower pattern is present, though at a slower rate compared to patterns reported in other Nordic or European countries. Also the deceleration in immunity acquisition during the primary school (from 6 to 13 years of age) is a phenomenon observed in other countries, e.g., England and Wales, Germany, and Luxembourg [2,39], even though not to such a great extent as found in Norway. Indeed, the lower seroprevalence estimated by the mixture model among school age children older than 10 years in Norway is also a consequence of the reclassification of inconclusive cases, excluded by the traditional cut-off approach, among seronegative cases. Considering that most of the available analyses of serological data for VZV in Europe are based on the cut-off approach [39], it would be worth investigating whether the application of the mixture modelling approach to VZV data from other countries can detect a similar pattern.

Our results show that there is a non-negligible proportion of susceptible persons among adults, who have a higher risk of developing serious varicella and associated sequelae. Also, the risk of congenital varicella syndrome for the newborns is of concern, considering that almost half of the non-immune individuals of childbearing age are women. Moreover, we estimated a moderate, but prolonged, increase of the FOI to a plateau during the entire childbearing period (20–45 years), which might imply that, at these ages, contact with children is fundamental for maintaining the natural boosting of immunity. These findings are a key step towards the definition and calibration of the mathematical tools able to provide guidelines for the national decision about the introduction of varicella vaccination in Norway. Because long-term effects of varicella vaccination are still unclear and also due to the fear of a predicted increase in the incidence of HZ, systematically predicted by mathematical models of VZV transmission, many European countries are hesitant to use varicella vaccination at a large scale. In Norway, considering the higher level of the FOI estimated for the entire childbearing period and the rapid decline thereafter, long-term effects of varicella immunisation should be seriously considered, as these could avert the positive effect of natural boosting due to contact between parents and children.

This study illustrates the benefits of using a mixture modelling approach to assess VZV epidemiology. From an epidemiological point of view, these models provide reliable and unbiased estimates of the seropositive proportions by age for infections in both pre- and post-vaccination era [22,23,28,29,40–43]. Moreover, the mixture models are not affected by handling inconclusive cases, typical of the standard cut-off approach, as all individuals are assigned to the component for which they show higher probability. With respect to the work of Vyse and colleagues [23] in the UK, which is the only other published study on varicella seroepidemiology analysed using mixture models, our analysis went beyond the estimation of the age-specific proportions seropositive in the sample, as it included an estimation of the population seroprevalence and FOI.

The main study design limitations are linked to the sampling strategy to obtain the serological data, as already noted before [26], such as using a convenience sampling and not being able to ascertain which samples were collected from individuals originating from regions that may have higher levels of VZV susceptibility [44].

Further research will make use of the seroprevalence and FOI estimates as necessary input data for the parameterisation of an age-structured dynamic transmission model to be used to evaluate the potential impact of different varicella and HZ vaccination scenarios. In particular, the age-specific proportions of seropositive individuals estimated by the mixture model will be combined with available social contact data for Norway [45], in order to provide estimates of varicella transmission in the country.

## Supporting Information

S1 Table Estimate of the force of infection in Norway. Posterior mean of the FOI by age, with 95% credible intervals.
(PDF)

S1 Text Methodological note. (PDF)
