# Supplementary material for: Estimating Age-Specific Immunity and Force of Infection of Varicella Zoster Virus in Norway Using Mixture Models
Source: PLoS One. 2016 Sep 30;11(9):e0163636. doi: 10.1371/journal.pone.0163636 (PMC5045180; doi:10.1371/journal.pone.0163636)
Supplement: S1 Table — Posterior mean of the FOI by age, with 95% credible intervals. (PDF) [file pone.0163636.s001.pdf]

S1 Table Estimate of the force of infection in Norway. Posterior mean of the FOI by age, with 95% credible intervals.

Age	FOI	95% CI
1y	0.104	0.067, 0.149
2y	0.174	0.110, 0.238
3y	0.263	0.160, 0.374
4y	0.233	0.121, 0.357
5y	0.263	0.108, 0.437
6y	0.139	0.030, 0.290
7y	0.116	0.022, 0.246
8y	0.091	0.013, 0.221
9y	0.053	0.007, 0.137
10-14y	0.048	0.006, 0.125
15-19y	0.042	0.005, 0.113
20-24y	0.039	0.005, 0.107
25-29y	0.044	0.005, 0.120
30-34y	0.052	0.007, 0.143
35-39y	0.053	0.007, 0.146
40-44y	0.057	0.007, 0.156
45-49y	0.057	0.007, 0.159
50-59y	0.044	0.005, 0.134
>60y	0.025	0.003, 0.080
