# Supplementary material for: Estimating Age-Specific Immunity and Force of Infection of Varicella Zoster Virus in Norway Using Mixture Models
Source: PLoS One. 2016 Sep 30;11(9):e0163636. doi: 10.1371/journal.pone.0163636 (PMC5045180; doi:10.1371/journal.pone.0163636)
Supplement: S1 Text — (PDF) [file pone.0163636.s002.pdf]

S1 Text Methodological Note

In this supplementary text, we give more details about the estimation of the mixture model parameters, the seroprevalence function, and the age-specific proportions of seropositive individuals.

Estimation of the mixture model for antibody data

Definition of the mixture model for pre-vaccination antibody data

We estimated a two-component hierarchical Bayesian mixture model [1], allowing for component-specific mixture parameters. The choice of a two-component mixture is motivated by the fact that the infection under study is in a pre-vaccination equilibrium, thus we do not expect a large heterogeneity in the antibody counts of the seropositive individuals, as it occurs with post-vaccination infections [2,3]. The first component is the distribution of the antibody counts of the susceptible and the second component is the distribution of the antibody counts of the immune. Assuming that mixture weights are dependent on age, the mixture model can be written as

$$g(Y_i(a)) = \sum_{k=1}^{2} \pi_k(a) f_k(Y_i(a)|\boldsymbol{\xi}_i),$$

where $Y_i = \log_{10}(OD_i + 1)$ is the individual antibody count, a denotes individuals' chronological age, $f(Y_i|\boldsymbol{\xi}_k), k = 1,2,$ are the mixture components of the model with density function f, $\pi_k(a), k = 1,2,$ are the age-dependent mixture weights of each component, and, finally, $\xi_k,\ k = 1,2,$ are the vectors of parameters to estimate [4].

In order to assign each individual to one of the components, based on his antibody count, we introduce a latent age-dependent indicator variable $Z_i(a)$, which represents the unknown infection status of individual with age a [5], and has the following Bernoulli distribution:

$$Z_i(a) = \begin{cases} 1 & \text{with probability } \pi(a) & \text{seropositive,} \\ 0 & \text{with probability } 1 - \pi(a) & \text{seronegative.} \end{cases}$$

This classification variable has the same meaning of the current status data determined by the cut-off approach. The probabilities associated with the two events, $\pi(a)$ and $1 - \pi(a)$, are the mixing weights of the immune and the susceptible components, respectively, and govern the assignment of the individual cases to each of the components. In particular, $\pi(a)$ is the probability that an individual in the population belongs to the immune component and can be interpreted as the seroprevalence in the population [6].

Estimation of the mixture parameters

Different densities for the distribution of the data can be taken into account to model the data. An obvious choice is the Normal distribution [7], which is a reasonable assumption mostly for the susceptible component. However, since it has been reported that, for some infections, the immune component might be characterized by skewness (longer tails), other distributions that allow for it should be considered [8,9]. Possible distributions are, for instance, the Skew-Normal distribution, which allows for skewness, the Student's t distribution, which allows for thicker tails, or the Skew-t distribution, which accounts for both deviations from normality [10]. Hereafter, we focus on the Skew-Normal distribution, used to model the antibodies in the main manuscript, as data inspection reveals some degree of positive skewness in the immune component.

The Skew-Normal distribution [11] is an extension of the Normal distribution that allows for skewness in the data. The probability density function (pdf) of this distribution is given by

$$f(X|\mu, \sigma^2, \alpha) = \frac{2}{\sigma}\phi\left(\frac{X-\mu}{\sigma}\right)\Phi\left(\alpha\frac{X-\mu}{\sigma}\right)$$

where ϕ and Φ are the pdf and the cumulative density function of the standard

normal distribution, respectively. The parameters μ and σ are the location and the scale parameters, respectively, and α is the skewness parameter, which can lead to a skewness coefficient in the interval [-0.9953, 0.9953].

In order to fit a Bayesian mixture model with skew-normally distributed components, we use a stochastic representation of the distribution, based on a random-effect model [10]. We define the variable $Y = \mu + \sigma\delta S + \sigma\sqrt{1-\delta^2}\varepsilon$, where S is a random effect with truncated Normal distribution, $S \sim TN_{[0,\infty]}(0,1)$, ε is the measurement error with Normal distribution, $\varepsilon \sim N(0,1)$, independent from S, and $\delta = \alpha/\sqrt{1+\alpha^2}$. In order to implement the Bayesian approach, the parameter vector $\boldsymbol{\theta}_k = (\mu_k, \sigma_k\delta_k, \sigma_k\sqrt{1-\delta_k^2})$ is parameterised as $\boldsymbol{\theta}_k^* = (\mu_k, \psi_k, \omega_k)$ [10]. Hence, the skew-normal mixture model for $Y_i(a)$ can be rewritten as a normal mixture model with the following parameters:

$$g(Y_i(a)) = \sum_{k=1}^{2} \pi_k(a) N(Y_i(a) | \mu_k + \psi_k S_i, \omega_k^2).$$

The parameters σ_k^2 and α_k can be recovered through $\sigma_k^2 = \omega_k^2 + \psi_k^2$ and $\alpha_k = \psi_k/\omega_k$. For the prior distributions of the parameters μ_k, ω_k, and ψ_k, we choose the following flat distributions [5]:

$$\mu_k \sim N(m_k, \tau_k/\zeta_k), \text{ with } \mu_1 \leq \mu_2;$$

$$\tau_k = 1/\omega_k^2 \sim U(0,1000);$$

$$\psi_k \sim N(0,100).$$

Finally, for the hyperparameters $m_k$and ζ_k, we choose the following flat distributions:

$$m_k \sim N(0,1000);$$

$$\zeta_k \sim \Gamma(0.001,0.001).$$

Estimation of the seroprevalence and FOI

The seroprevalence and the FOI function are estimated simultaneously with the mixture parameters and with the latent classification variables $Z_i(a)$.

As prior distribution for the age-specific seroprevalence $\pi_k(a)$, we choose a beta distribution with age-specific parameters, namely, $\pi_j \sim \text{Beta}(\alpha_j, \beta_j)$, where $j = 1,2,\dots,n$ denotes the jth age group. Since the seroprevalence model ought to guarantee a nonnegative FOI, we must constrain it to be monotonically increasing. This is accomplished by using the monotonicity constraint $\pi_{j-1} \leq \pi_j \leq \pi_{j+1}$, which performs a smoothing, averting cases where the seroprevalence first increases and then decreases (or vice versa).

Combining the given beta prior distribution for π_j, $\pi_j \sim \text{Beta}(\alpha_j, \beta_j)$, with the binomial data, $X_j \sim \text{Bin}(\pi_j, n_j)$ (given by the seropositive results according to the latent data $Z_i(a)$, aggregated by age group, $X_j = \sum_{i=1}^{N} I_{Z_i(a_j)=1}$), it follows that the posterior distribution of the seroprevalence in age group j is again a beta distribution, $\pi_j | X_j \sim \text{Beta}(X_j + \alpha_j, n_j - X_j + \beta_j)$, under the same monotonicity constraint, $\pi_{j-1} \leq \pi_j \leq \pi_{j+1}$.

Finally, we estimate the FOI by the following formula:

$$\lambda_j = \pi_j' / (1 - \pi_j) \cong [(\pi_{j+1} - \pi_{j-1})/2] \,/\, (1 - \pi_j).$$

Estimation of the proportions seropositive

After having obtained the estimates of the mixture parameters and of the classification variable, $\bar{Z}_i(a)$, obtained as posterior means from the posterior distribution of the parameters, we need to classify the components either as susceptible or as immune, and then assign the individuals to one of the two components.

First, according to the estimated location parameters μ_k, we label each component either as susceptible or immune: the component with the higher value of μ_k will be labelled as the "immune" component, the other one as the "susceptible" component.

Second, we assign each subject to the component for which the posterior mean of its classification variable is larger than 0.5: this means that each observation is assigned to the component for which it has the higher probability of belonging.

Finally, when each individual has been classified either as susceptible or immune, one can estimate the proportions seropositive per age group in a similar way to what it is done with the binary data obtained through the cut-off approach, i.e., by dividing the number of individuals assigned to the immune component $(\bar{Z}_i(a) = 1)$ in each age group for the total number of individuals in the age group.

References

1. Gelman A, Carlin JB, Stern HS, Dunson DB, Vehtari A, Rubin DB. Bayesian Data Analysis. third edition. CRC Press; 2014.

2. Rota MC, Massari M, Gabutti G, Guido M, De Donno A, Ciofi degli Atti ML. Measles serological survey in the Italian population: Interpretation of results using mixture model. Vaccine. 2008;26: 4403–4409. doi:10.1016/j.vaccine.2008.05.094

3. Del Fava E, Shkedy Z, Bechini A, Bonanni P, Manfredi P. Towards measles elimination in Italy: Monitoring herd immunity by Bayesian mixture modelling of serological data. Epidemics. Elsevier B.V; 2012;4: 124–131. doi:10.1016/j.epidem.2012.05.001

4. McLachlan G, Peel D. Finite Mixture Models. John Wiley & Sons; 2004.

5. Diebolt J, Robert CP. Estimation of finite mixture distributions through Bayesian sampling. J R Stat Soc Ser B Stat Methodol. JSTOR; 1994;56: 363–375.

6. Evans RB, Erlandson K. Robust Bayesian prediction of subject disease status and population prevalence using several similar diagnostic tests. Statist Med. 2004;23: 2227–2236. doi:10.1002/sim.1792

7. Parker RA, Erdman DD, Anderson LJ. Use of mixture models in determining laboratory criterion for identification of seropositive individuals: application to parvovirus B19 serology. J Virol Methods. 1990;27: 135–144.

8. Gay NJ. Analysis of serological surveys using mixture models: application to a survey of parvovirus B19. Statist Med. 1996;15: 1567–1573. doi:10.1002/(SICI)1097-0258(19960730)15:14<1567::AID-SIM289>3.0.CO;2-G

9. Vyse AJ, Gay NJ, Hesketh LM, Morgan-Capner P, Miller E. Seroprevalence of antibody to varicella zoster virus in England and Wales in children and young adults. Epidemiol Infect. 2004;132: 1129–1134. doi:10.1017/S0950268804003140

10. Frühwirth-Schnatter S, Pyne S. Bayesian inference for finite mixtures of univariate and multivariate skew-normal and skew-t distributions. Biostatistics. 2010;11: 317–336. doi:10.1093/biostatistics/kxp062

11. Azzalini A. A class of distributions which includes the normal ones. Scand J Stat. 1985;12: 171–178. doi:10.2307/4615982
